# NK Receptors: Tools for a Polyvalent Cell Family

**DOI:** 10.3389/fimmu.2014.00617

**Published:** 2014-12-03

**Authors:** Simona Sivori, Daniel Olive, Miguel López-Botet, Massimo Vitale

**Affiliations:** ^1^Dipartimento di Medicina Sperimentale (DI.ME.S.), Centro di Eccellenza per le Ricerche Biomediche (CEBR), Università degli Studi di Genova, Genova, Italy; ^2^U1068, CRCM, Immunity and Cancer, INSERM, Marseille, France; ^3^Institut Paoli-Calmettes, Marseille, France; ^4^UM 105, Aix-Marseille Université, Marseille, France; ^5^UMR7258, CNRS, Marseille, France; ^6^Hospital del Mar Medical Research Institute (IMIM), Universitat Pompeu Fabra, Barcelona, Spain; ^7^IRCCS Azienda Ospedaliera Universitaria S.Martino-IST, Genova, Italy

**Keywords:** KIR, NCR, anti-viral immune responses, anti-tumor immunity, innate immunity, ILC, autoimmune diseases, decidual NK cell

In these last years, along with the description of new NK cell types playing key roles within different body compartments, it is becoming conceivable that NK cell receptors might have been shaped to fulfill a complex array of functions beyond the simple recognition of pathological cells (1, 2). Such a process of adaptation to multiple needs offers new unexpected viewpoints on the evolution of NK receptors and their possible exploitation in a growing range of diseases. The first article of the present Research Topic will introduce this concept by providing a general discussion on how the major groups of receptors can be used by NK cells inside or outside the classical natural killing function (3). The following articles will go into detail of the various NK cell functions and the involved NK cell subsets, primarily considering the pressure exerted by physiological or pathological stimuli on the related receptors and their specific ligands.

Li and Mariuzza (4) describe the crystal structures of several surface receptors, belonging to the most known NK receptor families, and explain how such information could be critical for the prediction of possible molecular interactions with either extracellular ligands or intracellular signaling molecules. Cassidy et al. (5) focus on the KIR ligands, the MHC-I molecules, and analyze the role of the MHC-I peptide content in such receptor/ligand interaction suggesting that also NK cells can perceive in some ways intracellular modifications in tumor or virally infected cells.

Whether the peptide could also have a role in the HLA-I recognition by activating KIRs (aKIRs) is still unclear. Nevertheless, aKIRs are likely to play a role in infectious diseases, cancer, and autoimmunity. For example, aKIRs-mediated recognition of viruses and consequent NK cell priming has been proposed to occur and to be directly involved in the pathogenesis of the chronic lymphoproliferative disorder of NK cells (CLPD-NK) (6). Remarkably, several CLPD-NK cases present an expansion of aKIR^+^ NK cells, which are, nevertheless, characterized by reduced NCR-mediated function. In this context, aKIRs have been recently shown to participate in the education of NK cells, rendering them hyporesponsive. Interestingly, education by aKIRs shares features with the hyporesponsiveness induced by chronic stimulation of other activating receptors expressed by NK cells (7).

All the above mentioned articles can provide hints on the process of co-evolution that may have affected KIRs, their autologous ligands and exogenous molecules or peptides, as well as the mechanisms controlling the strength of NK cell response to stimuli. A possible receptor/ligand co-evolution may have also concerned several non-MHC-specific activating receptors. A living recapitulation of how this co-evolution may have occurred is represented by the struggle between NK cells and tumors. Cerboni et al. (8) and Huergo-Zapico et al. (9) describe different molecular pathways that can convey stress or damage signals to enhance the expression of various ligands of NKG2D or DNAM-1 activating receptors. These signals are often related to the process of tumor transformation. Bottino et al. (10), Baier et al. (11), Chouaib et al. (12), and Chretien et al. (13) show that a large part of the non-MHC-specific activating NK receptors can recognize ligands overexpressed on tumors. On the other hand, they also show that really many strategies are executed by the tumor to avoid the NK cell attack. In the end, the authors indicate how a deeper understanding of the forces involved in the NK/tumor struggle is essential to develop more effective NK cell-based immunotherapy against cancer.

Remarkably, the quantitative deficiency of NK cells or the altered expression/function of their receptors witness the negative influence exerted not only by tumor microenvironment but also by viruses. Lugli et al. (14) and Marras et al. (15) show that viruses (i.e., HIV, HCV, and hCMV) are able to affect the functional status and the homeostasis of NK cell population through the modulation/engagement of several surface receptors and the expansion of unconventional poorly functional NK cell subsets.

As above mentioned, because of their polyvalent role, certain receptors may have been exposed to multiple shaping pressures. For example, NK cells that populate decidua (dNK) in the first trimester of pregnancy can use NKp44, NKp30, and KIR2DL4 both to control infections and to promote regulatory interactions [with trophoblasts or dendritic cell (DC)] important for placental development (16). The abundance of dNK cells in the decidua in a specific phase of pregnancy suggests that these cells could be determinant for a safe and successful childbirth, indicating that reproduction may have significantly intervened on the evolution of certain NK receptors. The dual role of certain NK receptors and the promiscuity of their ligands have also been recently highlighted by the characterization of the large family of innate lymphoid cells (ILCs). Cella et al. (17) and Killig et al. (18) discuss this issue. ILCs play a relevant role in the defense against invading microbes, but also in tissue remodeling including the induction of lymphoid tissues as well as the homeostasis of epithelial barrier in the mucose. They include two different NK-R^+^ cell types: the conventional NK cells (belonging to group 1 ILCs) and the IL22-producing NCR^+^ILC3 cells (belonging to group 3 ILCs). Depending on the environmental mediators, engagement of NKp44 and PRRs can mediate release of the ILC3 signature cytokine IL-22 or pro-inflammatory soluble factors thus modulating both epithelial and immune cell functions at mucosal interfaces.

Also, NKp30 can fulfill different tasks, as it can serve as a tool for eliminating altered cells and promoting regulatory interaction with autologous cells. Reiners et al. (19) shows that exosomes derived from both tumor cells and activated DCs can play a relevant role in the regulation of NK cell function. Interestingly, in both cases, interaction of NKp30 with its ligand BAG6 on exosomes can activate NK cells.

Different studies have highlighted the important immunomodulatory role of the crosstalk between NK cells and DCs. Ferlazzo and Morandi (20) describe the new DC subsets recently identified in the human system and suggest that the NK/DC interactions should be considered as a complex network of cell subset cooperation acting in discrete regions of the body to fulfill complementary tasks.

The functional interaction with normal cells (such as DCs or trophoblasts) implies that several NK receptors can recognize autologous ligands. These considerations indicate that a potential role for NK cells also in the development of autoimmune diseases. Poggi and Zocchi (21) and Enk and Mandelboim (22), respectively, describe how the inappropriate interaction with APC or MSC and the ectopic/abnormal expression of certain NCR-ligands could be implied on several autoimmune diseases thus contributing to widen the range of pathologies potentially involving NK cells.

In conclusion, the analysis of the NK cell functions and receptors as discussed in this Research Topic indicates that, in spite of the continuous flow of discoveries that have significantly changed key concepts on the biology of NK cells, “the recognition of self,” originally postulated by Kärre more than 20 years ago as strategy to drive NK cell cytotoxicity (23), still represents the basis of NK cell function. Exactly following this core strategy, NK cells have evolved their wide range of receptors required to fulfill their ever increasing number of functions. NK receptors mostly recognize autologous ligands whose expression could be induced/regulated by stress, viral infection, cell damage, or activating stimuli. Through these interactions, NK receptors regulate the response to pathogens or dangerous altered cells, but also take part to functional interactions with different elements of peripheral tissues.

## Conflict of Interest Statement

The authors declare that the research was conducted in the absence of any commercial or financial relationships that could be construed as a potential conflict of interest.
